# SoDCoD: a comprehensive database of Cu/Zn superoxide dismutase conformational diversity caused by ALS-linked gene mutations and other perturbations

**DOI:** 10.1093/database/baae064

**Published:** 2024-08-08

**Authors:** Riko Tabuchi, Yurika Momozawa, Yuki Hayashi, Hisashi Noma, Hidenori Ichijo, Takao Fujisawa

**Affiliations:** Laboratory of Cell Signaling, Graduate School of Pharmaceutical Sciences, The University of Tokyo, 7-3-1 Hongo, Bunkyo-ku, Tokyo 113-0033, Japan; Laboratory of Cell Signaling, Graduate School of Pharmaceutical Sciences, The University of Tokyo, 7-3-1 Hongo, Bunkyo-ku, Tokyo 113-0033, Japan; Laboratory of Cell Signaling, Graduate School of Pharmaceutical Sciences, The University of Tokyo, 7-3-1 Hongo, Bunkyo-ku, Tokyo 113-0033, Japan; Department of Data Science, The Institute of Statistical Mathematics, 10-3 Midori-cho, Tachikawa, Tokyo 190-8562, Japan; Laboratory of Cell Signaling, Graduate School of Pharmaceutical Sciences, The University of Tokyo, 7-3-1 Hongo, Bunkyo-ku, Tokyo 113-0033, Japan; Laboratory of Cell Signaling, Graduate School of Pharmaceutical Sciences, The University of Tokyo, 7-3-1 Hongo, Bunkyo-ku, Tokyo 113-0033, Japan

## Abstract

A structural alteration in copper/zinc superoxide dismutase (SOD1) is one of the common features caused by amyotrophic lateral sclerosis (ALS)–linked mutations. Although a large number of SOD1 variants have been reported in ALS patients, the detailed structural properties of each variant are not well summarized. We present SoDCoD, a database of superoxide dismutase conformational diversity, collecting our comprehensive biochemical analyses of the structural changes in SOD1 caused by ALS-linked gene mutations and other perturbations. SoDCoD version 1.0 contains information about the properties of 188 types of SOD1 mutants, including structural changes and their binding to Derlin-1, as well as a set of genes contributing to the proteostasis of mutant-like wild-type SOD1. This database provides valuable insights into the diagnosis and treatment of ALS, particularly by targeting conformational alterations in SOD1.

**Database URL**: https://fujisawagroup.github.io/SoDCoDweb/

## Introduction

Amyotrophic lateral sclerosis (ALS) is a fatal neurodegenerative disorder with no cure. Copper/zinc (*Cu/Zn*) *Superoxide Dismutase* (*SOD1*) is one of the causative genes for ALS, and over 180 distinct mutations accompanied by amino acid changes have been reported in ALS patients [1–3]. However, the common molecular mechanisms as to why different types of *SOD1* gene mutations cause ALS are not yet fully understood.

We previously reported that most SOD1 mutants (SOD1^mut^) bind to the carboxyl-terminal region of Derlin-1 [termed Derlin-1(CT4)], a component of the endoplasmic reticulum–associated degradation machinery, and this interaction triggers endoplasmic reticulum stress–dependent motor neuron death [4]. In the process of this research, we identified 5–18 amino acids of SOD1 as the Derlin-1 binding region (DBR) [5]. In order to assess whether the SOD1^mut^ undergoes any structural changes that expose the DBR, we developed antibodies with an epitope at or near the DBR. As a result, we succeeded in developing MS785, which has an epitope of 6–16 amino acids, a part of the DBR, and MS27, which has an epitope of 30–40 amino acids near the DBR [5, 6]. These antibodies were found to recognize all SOD1^mut^ that bind to Derlin-1, indicating that most SOD1^mut^ exhibit ER stress–induced motor neuron toxicity. On the other hand, some *SOD1* mutations did not induce structural changes that are recognized by these antibodies, suggesting that they may have distinct mechanisms of exerting toxicity or even be single-nucleotide polymorphisms that are not linked to ALS pathogenesis. Therefore, there is a need to understand the unique characteristics of each SOD1^mut^ in order to develop a therapeutic strategy for ALS tailored to each *SOD1* mutation.

Although still controversial, there are several reports indicating the involvement of wild-type SOD1 (SOD1^WT^) in the pathogenesis of *SOD1* mutation–negative ALS. A mutant-like conformational change in SOD1^WT^ was observed in samples derived from *SOD1* mutation–negative sporadic ALS patients [7, 8]. In addition, we reported that zinc deficiency induces a conformational alteration in SOD1^WT^ [9]. These reports suggest that a cellular stress–dependent defect in SOD1^WT^ proteostasis might potentially contribute to ALS pathogenesis. To understand the role of SOD1^WT^ in the pathogenesis of *SOD1* mutation-negative ALS, we performed a genome-wide small interfering RNA (siRNA) screening and found candidate genes involved in the proteostasis of mutant-like SOD1^WT^ [10]. Information about these genes would be helpful in improving our understanding of the involvement of SOD1^WT^ in the pathogenesis of ALS.

Here, we present SoDCoD version 1.0, a comprehensive database of Cu/Zn superoxide dismutase conformational diversity caused by ALS-linked gene mutations and other perturbations. This version contains information about the properties of SOD1 caused by 188 distinct mutations, including structural changes and their binding to Derlin-1, as well as a set of genes contributing to the proteostasis of mutant-like SOD1^WT^. This database provides indispensable information for significant progress in medical research, diagnosis, and treatment of ALS, targeting conformational alterations in SOD1.

## Materials and methods

### Plasmids

Plasmids for expressing FLAG-SOD1^WT^, FLAG-SOD1^G93A^, and Venus-Derlin-1(CT4)-HA in pcDNA3.0 were previously constructed [4]. Other SOD1^mut^ plasmids in pcDNA3.0 were prepared by polymerase chain reaction-mediated site-directed mutagenesis. A complete list of primers for mutagenesis is provided in Supplementary Table S1.

### Cell culture and transfection

HEK293 cells were cultured in Dulbecco’s Modified Eagle’s Medium (Sigma-Aldrich, D5796) containing 10% fetal bovine serum (Gibco, 10270-106) and 100 units/ml penicillin G (Meiji Seika, 01028-85) in 5% CO_2_ at 37°C. Plasmid transfection was performed using Polyethylenimine “MAX” (Polysciences, 24765) according to the manufacturer’s instructions.

### Immunoprecipitation analysis

Cells were lysed in a buffer containing 20 mM Tris-HCl, pH 7.5, 150 mM NaCl, 10 mM ethylenediaminetetraacetic acid, 1% Triton X-100, 5 µg/ml leupeptin, and 1 mM phenylmethylsulfonyl fluoride. For immunoprecipitation (IP) with anti-FLAG antibody, cell lysates were incubated with anti-DYKDDDDK tag Antibody Beads (Wako, 016-22784) at 4°C for 10 min. For IP with MS785-MS27 or MS27 (MS antibodies), cell lysates were incubated with the antibodies at 4°C for 16 h and then incubated with the protein G-Sepharose (GE, 17-0618-02) for 1 h at 4°C. The beads were washed with washing buffer 1 containing 20 mM Tris-HCl, pH 7.5, 500 mM NaCl, 5 mM ethylene glycol-bis(β-aminoethyl ether)-N,N,N′,N′-tetraacetic acid (EGTA), and 1% Triton X-100 and washing buffer 2 containing 20 mM Tris-HCl, pH 7.5, 150 mM NaCl, and 5 mM EGTA, separated by sodium dodecyl sulfate–polyacrylamide gel electrophoresis and immunoblotted with antibodies to hemagglutinin (Roche, 11867431001) or FLAG (Wako, 012-22384). The proteins were detected by the enhanced chemiluminescence system (Cytiva, GERPN2235).

### Web interface

The web interface was coded in HTML, CSS, and JavaScript with the Bootstrap framework. The curated data were transformed into the HTML format and can be accessed through a user-friendly web interface. The graphical user interface was tested in Google Chrome with WebGL implemented.

## Data collection

### Classification of all the SOD1^mut^

**Figure 1.**
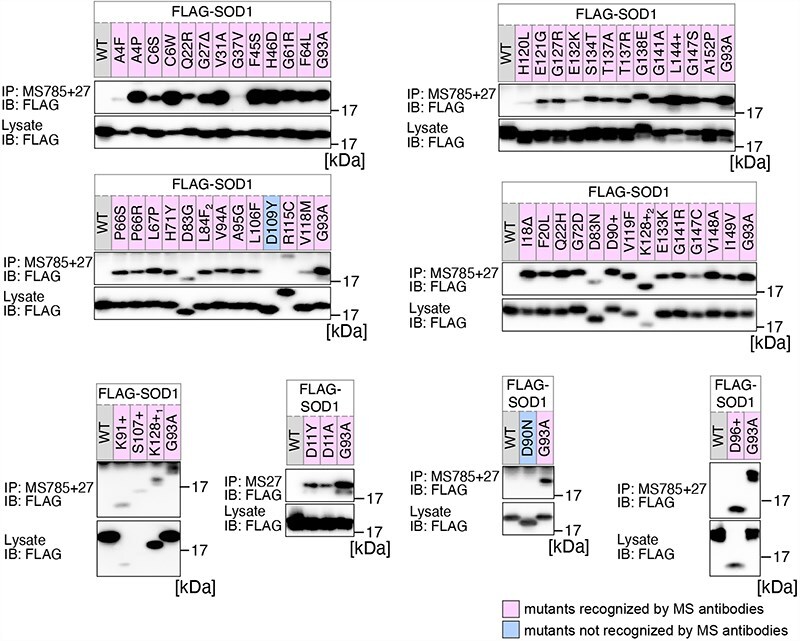
Analysis of the structural changes in 56 variants of SOD1. HEK293 cells were transfected with various FLAG-SOD1 mutants. Lysates from the transfected cells were analyzed by IP and immunoblotting (IB) with the indicated antibodies. +, insertion; ∆, deletion; L84F_2_, L84(TTG) to F(TTT); K128+_1_, c.383_384insACCC; K128+_2_, c.384_385insTGGG.

**Figure 2.**
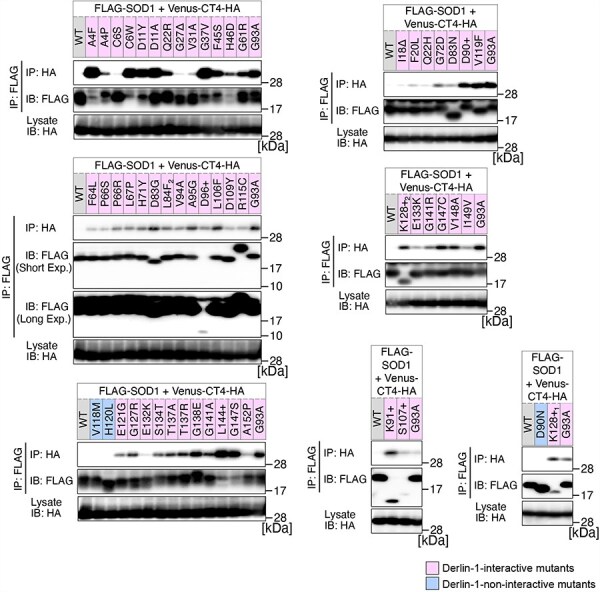
Analysis of the binding of 56 variants of SOD1 to Derlin-1(CT4). HEK293 cells were co-transfected with pcDNA3.0-variant of yellow fluorescent protein (Venus)-Derlin-1(CT4)-HA and various FLAG-SOD1 mutants. Lysates from the transfected cells were analysed by IP–IB with the indicated antibodies. +, insertion; ∆, deletion; L84F_2_, L84(TTG) to F(TTT); K128+_1_, c.383_384insACCC; K128+_2_, c.384_385insTGGG. Short Exp., Short exposure; Long Exp., Long exposure; IB, immunoblotting.

**Figure 3.**
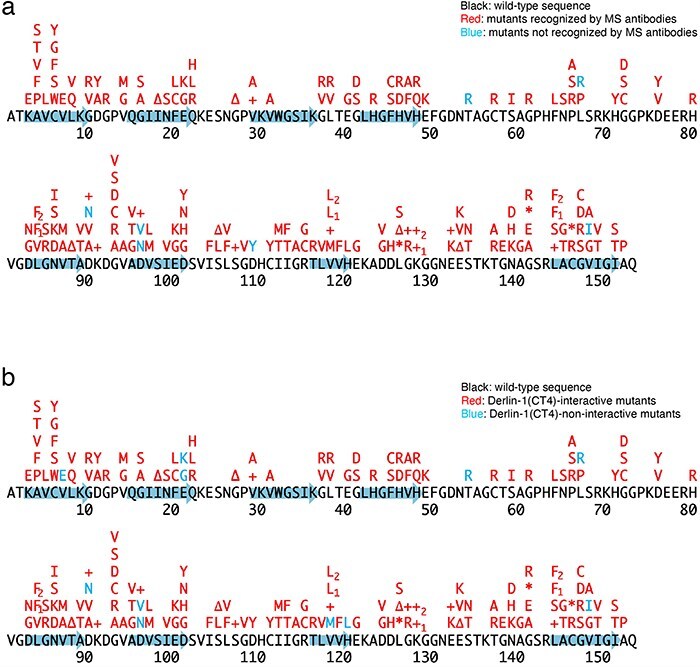
Schematic diagrams of the 188 SOD1 mutants. +, insertion; ∆, deletion; *, nonsense mutation; arrows, the beta-strands. L84F_1_, L84(TTG) to F(TTC); L84F_2_, L84(TTG) to F(TTT); K128+_1_, c.383_384insACCC; K128+_2_, c.384_385insTGGG; V118L_1_, V118(GTG) to L(CTG); V118L_2_, V118(GTG) to L(TTG); L144F_1_, L144(TTG) to F(TTC); L144F_2_, L144(TTG) to F(TTT). (a) Conformational alterlation in SOD1 mutants. (b) SOD1 mutants-Derlin-1(CT4) interaction.

Previously, we conducted a comprehensive analysis of 132 SOD1^mut^ concerning their structural alterations and interaction with Derlin-1 [5, 6]. Subsequently, as of May 2022, an additional 56 novel genetic variants were recorded in the ALSoD database [1, 2]. In this study, we performed a functional classification of the 56 SOD1^mut^. As a result, 53 SOD1^mut^ bind to Derlin-1(CT4), and 54 SOD1^mut^ exhibit notable conformational changes (Figs. 1 and 2). Taken together with our previous reports, 177 SOD1^mut^ out of 188 were classified as Derlin-1-interactive SOD1^mut^ and 181 SOD1^mut^ as aberrant in conformation (Fig. 3A and B).

We acknowledge that the degree of recognition by our antibodies varied depending on the types of *SOD1* gene mutations. Thus, the band intensities obtained through western blots were quantified using Fiji software [11]. The band intensities of each SOD1^mut^ immunoprecipitated by MS785 and MS27 were normalized against the band intensity of SOD1^mut^ in the lysate. The band intensity of each SOD1^mut^ was then normalized as relative values with the band intensity of SOD1^G93A^ set to 1.0.

### Other properties of SOD1^mut^

Numerous biochemical studies have been carried out on each SOD1^mut^, which have revealed their Cu/Zn binding affinity, enzyme activity, and crystal structures. To facilitate an investigation of the interplay between these properties of the SOD1^mut^ and their binding to Derlin-1 and conformational changes, we decided to gather this information in the database. The Cu/Zn coordination and enzyme activity of each SOD1^mut^ were obtained through a comprehensive literature search. In addition, all structural information was obtained from the Protein Data Bank [12].

### Regulators of mutant-like SOD1^WT^

We previously performed an siRNA screening to identify modulators of conformational alteration in SOD1^WT^ and reported that DDB1 and CUL4 Associated Factor 4 governs proteostasis of mutant-like SOD1^WT^ [10]. In the present study, we cataloged other candidate genes that were positive in the screen within the database.

## Use cases

### Web interface

**Figure 4.**
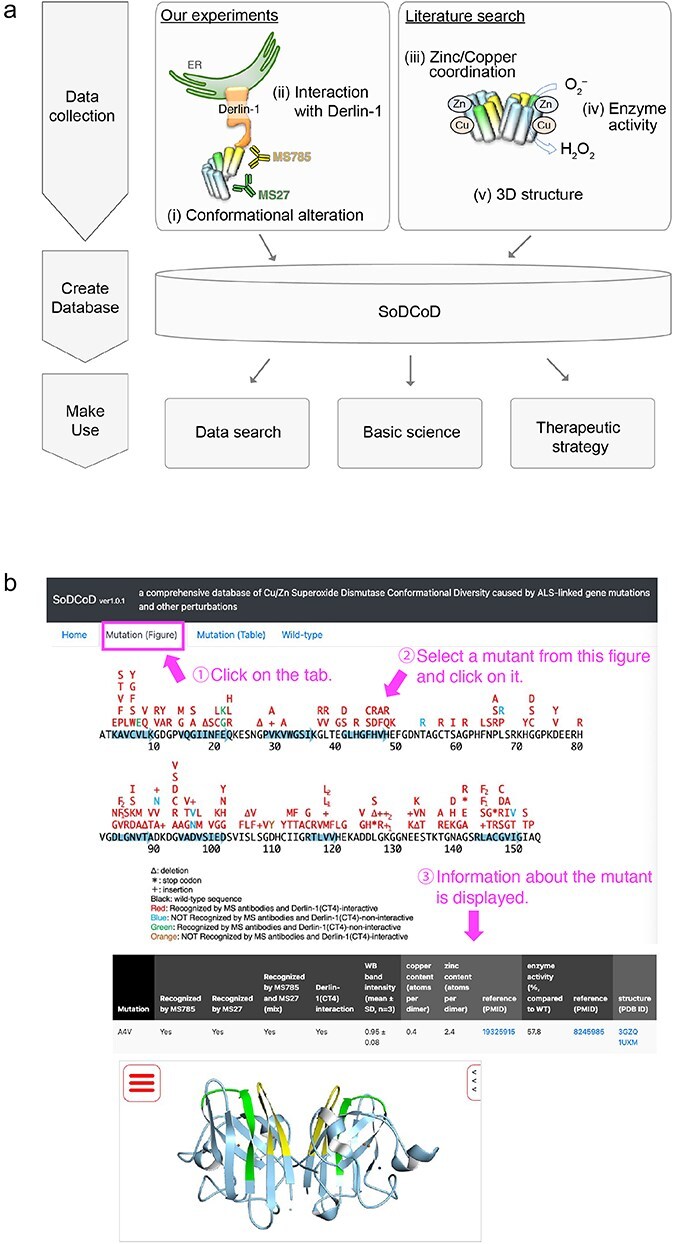
Screenshots of the SoDCoD web page. (a) A database schema. (b) An example usage of the database. By clicking on a mutation in the image, the results of the biochemical analysis are displayed in the table.

The graphical interface of the SoDCoD database version 1.0 contains four tab panels: (i) Home, (ii) Mutation (Figure), (iii) Mutation (Table), and (iv) Wild-type. The “Home” tab contains a general description of the database and how to use it. The “Mutation (Figure)” tab is the main content of this website, and by clicking on the *SOD1* gene mutation in the diagram, the results of the biochemical analysis and a 3D structure of SOD1 are displayed in the table (Fig. 4). The 3D structure is embedded by Molmil, a 3D molecular viewer [13]. The information on the mutants can also be obtained from the list of the mutations on the “Mutation (Table)” tab panel. The “Wild-type” tab contains the results of our siRNA screening to identify genes involved in the proteostasis of conformationally altered SOD1^WT^.

### An example usage

Recent advances in DNA sequencing technologies have revolutionized our understanding of genetic mutations and their roles in the pathogenesis of ALS. Once a mutation in *SOD1* has been identified in an ALS patient, it is generally recommended to follow the guidelines of the American College of Medical Genetics and Genomics to determine the pathogenicity of the mutation [14]. Nevertheless, due to the lack of experimental and clinical evidence, it is frequently infeasible to assert whether a genetic mutation detected by genetic testing is disease-causative. Our database provides comprehensive information about the structural abnormalities in SOD1^mut^ found in ALS patients. Hence, this comprehensive resource helps to determine the pathogenic nature of each *SOD1* mutation, thereby facilitating more precise and personalized medical interventions tailored to an individual’s unique genetic characteristics.

Recently, therapeutic strategies utilizing the structural characteristics of SOD1^mut^ have attracted much attention. We developed an inhibitor of the SOD1–Derlin-1(CT4) interaction that ameliorates ALS pathology in patient-derived induced pluripotent stem cell–derived motor neurons harboring an SOD1 mutation and in an ALS mouse model expressing a human SOD1 G93A mutant [15]. In addition, another research group presented evidence that a Derlin-1(CT4) peptide-based protein knockdown system induced the degradation of the SOD1^mut^, delayed disease onset, and prolonged lifespan in an ALS mouse model [16]. When considering the applicability of these disease treatment strategies, it is important to determine whether the specific mutations found in ALS patients are indeed a potential target for these therapies. The information in our database will offer useful perspectives on this matter.

Several reports have demonstrated the mechanisms of SOD1^mut^-induced motor neuron toxicity, including ER stress, mitochondrial toxicity, and neuroinflammation [4, 17, 18]. Currently, several drugs are in clinical trials to suppress these toxicities [19]. However, it is uncertain whether these drugs will be effective against ALS caused by all SOD1 mutations, as the studies have mainly been conducted with specific mutants (e.g. A4V, G85R, and G93A). In order to promote the future development of personalized medicine, it is important to take into account the mechanism of toxicity exerted by each SOD1^mut^. SoDCoD offers a summary of the biochemical properties of each SOD1^mut^, which is anticipated to be beneficial for basic research into the mechanism of toxic effects caused by each variant of SOD1.

## Conclusion and future development

Databases need to be updated frequently and constantly to provide up-to-date information. In this era of genomic sequencing, novel *SOD1* mutations should be continuously identified in ALS patients. To keep pace, we will regularly investigate the potential impact of novel SOD1 mutations on Derlin-1 binding and conformational changes, thereby updating our database.

In addition, we will conduct basic scientific research utilizing this database. Notably, the extent of Derlin-1 binding and conformational changes in SOD1 varied markedly among mutant types. Genetic mutations in *SOD1* have been reported to have different clinical manifestations depending on the mutation [20]. We are currently analyzing whether these features correlate with the clinical phenotypes of ALS (i.e. onset or survival) and are preparing to publish this as SoDCoD version 2.0. Basic scientific research utilizing this database will contribute to our understanding of the pathogenesis of ALS.

## Supplementary Material

baae064_Supp

## Data Availability

All data in this report are deposited on GitHub and available for researchers to access (https://github.com/FujisawaGroup/SoDCoD).
