# Supplementary material for: SoDCoD: a comprehensive database of Cu/Zn superoxide dismutase conformational diversity caused by ALS-linked gene mutations and other perturbations
Source: Database (Oxford). 2024 Aug 8;2024:baae064. doi: 10.1093/database/baae064 (PMC11315765; doi:10.1093/database/baae064)
Supplement: baae064_Supp [file baae064_supp.zip › suppl_data/Supplementary Table S1.docx]

| Primer | Sequence (5’-3’) |
| --- | --- |
| A152P_forward | GGTGTAATTGGGATCCCCCAATAA |
| A152P_reverse | TTATTGGGGGATCCCAATTACACC |
| A4F_forward | ATGGCGACGAAGTTCGTGTGCGT |
| A4F_reverse | TTCAGCACGCACACGAACTTCGTC |
| A4P_forward | ATGGCGACGAAGCCCGTGTGCGT |
| A4P_reverse | TCAGCACGCACACGGGCTTCGTC |
| A95G_forward | ACAAAGATGGTGTGGGCGATGTG |
| A95G_reverse | TCAATAGACACATCGCCCACACCA |
| C6S_forward | GCGACGAAGGCCGTGAGCGTGCT |
| C6S_reverse | CGCCCTTCAGCACGCTCACGGCCT |
| C6W_forward | GACGAAGGCCGTGTGGGTGCTGA |
| C6W_reverse | GTCGCCCTTCAGCACCCACACGGC |
| D109Y_forward | ATCTCACTCTCAGGATACCATTGC |
| D109Y_reverse | CAATGATGCAATGGTATCCTGAGA |
| D11A_forward | GCGTGCTGAAGGGCGCCGGCCCA |
| D11A_reverse | CCCTGCACTGGGCCGGCGCCCTT |
| D11Y_forward | TGCGTGCTGAAGGGCTACGGCCC |
| D11Y_reverse | CCTGCACTGGGCCGTAGCCCTTCA |
| D83G_forward | AGAGGCATGTTGGAGGCTTGGGC |
| D83G_reverse | GTCACATTGCCCAAGCCTCCAACA |
| D83N_forward | TTGGAAACTTGGGCAATGTGA |
| D83N_reverse | CAAGTTTCCAACATGCCTCTC |
| D90N_forward | CTGCTAACAAAGATGGTGTGG |
| D90N_reverse | TTTGTTAGCAGTCACATTGCC |
| D90+_forward | TGACAACAAAGATGGTGTGGCCG |
| D90+_reverse | ATCTTTGTTGTCAGCAGTCACAT |
| D96+_forward | AAAGATGGTGTGGCCATGTGTCTA |
| D96+_reverse | CTTCAATAGACACATGGCCACACC |
| E121G_forward | CACTGGTGGTCCATGGAAAAGCA |
| E121G_reverse | AAGTCATCTGCTTTTCCATGGACC |
| E132K_forward | GGCAAAGGTGGAAATAAAGAAAG |
| E132K_reverse | TCTTTGTACTTTCTTTATTTCCACC |
| E133K_forward | ATGAAAAAAGTACAAAGACAG |
| E133K_reverse | ACTTTTTTCATTTCCACCTTT |
| F20L_forward | AATTTGGAGCAGAAGGAAAGT |
| F20L_reverse | TGCTCCAAATTGATGATGCCC |
| F45S_forward | AAGGCCTGCATGGATCCCATGTTC |
| F45S_reverse | AACTCATGAACATGGGATCCATGC |
| F64L_forward | AGTGCAGGTCCTCACCTTAATCCT |
| F64L_reverse | TGGATAGAGGATTAAGGTGAGGA |
| G127R_forward | AAAGCAGATGACTTGCGCAAAGG |
| G127R_reverse | CATTTCCACCTTTGCGCAAGTCAT |
| G138E_forward | AAAGTACAAAGACAGAAAACGCT |
| G138E_reverse | CGACTTCCAGCGTTTTCTGTCTTT |
| G141A_forward | AGACAGGAAACGCTGCAAGTCGT |
| G141A_reverse | CAAGCCAAACGACTTGCAGCGTTT |
| G141R_forward | ACGCTAGAAGTCGTTTGGCTT |
| G141R_reverse | ACTTCTAGCGTTTCCTGTCTT |
| G147S_forward | AGTCGTTTGGCTTGTAGTGTAATT |
| G147S_reverse | CGATCCCAATTACACTACAAGCCA |
| G147C_forward | CTTGTTGTGTAATTGGGATCG |
| G147C_reverse | TACACAACAAGCCAAACGACT |
| G27Δ_forward | CAGAAGGAAAGTAATGTGAAGGT |
| G27Δ_reverse | TCCCCACACCTTCACATTACTTTCC |
| G37V_forward | GGGGAAGCATTAAAGTACTGACT |
| G37V_reverse | AGGCCTTCAGTCAGTACTTTAATG |
| G61R_forward | GGCTGTACCAGTGCACGTCCTCAC |
| G61R_reverse | GATTAAAGTGAGGACGTGCACTG |
| G72D_forward | ACACGATGGGCCAAAGGATGA |
| G72D_reverse | GCCCATCGTGTTTTCTGGATA |
| H120L_forward | GCACACTGGTGGTCCTTGAAAAAG |
| H120L_reverse | TCATCTGCTTTTTCAAGGACCACC |
| H46D_forward | GGCCTGCATGGATTCGATGTTCAT |
| H46D_reverse | CAAACTCATGAACATCGAATCCAT |
| H71Y_forward | CCTCTATCCAGAAAATACGGTGGG |
| H71Y_reverse | CCTTTGGCCCACCGTATTTTCTGG |
| I149V_forward | GTGTAGTTGGGATCGCCCAAT |
| I149V_reverse | CCCAACTACACCACAAGCCAA |
| I18Δ_forward | CATCAATTTCGAGCAGAAGG |
| I18Δ_reverse | CAGTGCAGGGCATCAATTTC |
| K128+1_forward | TTGGGACCCCAAAGGTGGAAATGA |
| K128+1_reverse | CTTTGGGGTCCCAAGTCATCTGCT |
| K128+2_forward | TGGGCTGGGAAAGGTGGAAATGAA |
| K128+2_reverse | CCTTTCCCAGCCCAAGTCATCTGC |
| K91_forward | TGACAGATGGTGTGGCCGAT |
| K91_reverse | CATCTGTCAGCAGTCACAT |
| L106F_forward | GATTCTGTGATCTCATTCTCAGGA |
| L106F_reverse | AATGGTCTCCTGAGAATGAGATCA |
| L144+_forward | GCTGGAAGTCGTTTCGTTTAGCTT |
| L144+_reverse | TACACCACAAGCTAAACGAAACGA |
| L67P_forward | CTCACTTTAATCCTCCATCCAGAA |
| L67P_reverse | CCGTGTTTTCTGGATGGAGGATTA |
| L84F2_forward | GCATGTTGGAGACTTTGGCAATGT |
| L84F2_reverse | AGCAGTCACATTGCCAAAGTCTCC |
| P66R_forward | GTCCTCACTTTAATCGTCTATCCA |
| P66R_reverse | TGTTTTCTGGATAGACGATTAAAG |
| P66S_forward | GGTCCTCACTTTAATTCTCTATCCA |
| P66S_reverse | GTTTTCTGGATAGAGAATTAAAGT |
| Q22H_forward | GAGCACAAGGAAAGTAATGGA |
| Q22H_reverse | TCCTTGTGCTCGAAATTGATG |
| Q22R_forward | TCATCAATTTCGAGCGGAAGGAAA |
| Q22R_reverse | CCATTACTTTCCTTCCGCTCGAAAT |
| R115C_forward | CATTGCATCATTGGCTGCACACTG |
| R115C_reverse | GGACCACCAGTGTGCAGCCAATG |
| S107+_forward | TCACTTCTCAGGAGACCATTG |
| S107+_reverse | CTGAGAAGTGAGATCACAGAA |
| S134T_forward | GTGGAAATGAAGAAACTACAAAG |
| S134T_reverse | TTTCCTGTCTTTGTAGTTTCTTCAT |
| T137A_forward | GAAGAAAGTACAAAGGCAGGAAA |
| T137A_reverse | TTCCAGCGTTTCCTGCCTTTGTACT |
| T137R_forward | AAGAAAGTACAAAGAGAGGAAAC |
| T137R_reverse | CTTCCAGCGTTTCCTCTCTTTGTAC |
| V118M_forward | ATTGGCCGCACACTGATGGTCCAT |
| V118M_reverse | CTTTTTCATGGACCATCAGTGTGC |
| V119F_forward | TGGTGTTCCATGAAAAAGCAG |
| V119F_reverse | ATGGAACACCAGTGTGCGGCC |
| V148A_forward | TGGTGCAATTGGGATCGCCCA |
| V148A_reverse | CAATTGCACCACAAGCCAAAC |
| V31A_forward | ATGGACCAGTGAAGGCGTGGGGA |
| V31A_reverse | TTAATGCTTCCCCACGCCTTCACT |
| V94A_forward | CTGACAAAGATGGTGCGGCCGAT |
| V94A_reverse | ATAGACACATCGGCCGCACCATCT |
